# Social health insurance, healthcare utilization, and costs in middle-aged and elderly community-dwelling adults in China

**DOI:** 10.1186/s12939-018-0733-0

**Published:** 2018-02-02

**Authors:** Zhonghua Wang, Xiangjun Li, Mingsheng Chen, Lei Si

**Affiliations:** 1 School of Health Policy & Management, Nanjing Medical University, 101Longmian Avenue, Jiangning District, Nanjing, 211166 China; 2 Institute of Healthy Jiangsu Construction & Development, Nanjing, 211166 China; 3 School of Health Economics and Management, Nanjing University of Chinese Medicine, Nanjing, 210023 China; 4 Centre for the Health Economy, Macquarie University, Sydney, NSW Australia

**Keywords:** Social health insurance, Healthcare utilization, Middle-aged and elderly adults, China

## Abstract

**Background:**

Although many studies have analyzed health insurance worldwide, most focus on whole populations rather than specific vulnerable groups. There is a lack of studies that compare different schemes. This paper evaluates the impact of different types of social health insurance and other associated factors on healthcare utilization and costs among middle-aged and elderly Chinese adults.

**Methods:**

Data were obtained from a nationally representative middle-aged and elderly household survey, the China Health and Retirement Longitudinal Study, which was conducted in 2015. Middle-aged and elderly are defined as people who are ≥45 years. Descriptive statistics were used to show the prevalence of each variable. Both logistic and multiple linear regression models were used to evaluate the association between healthcare utilization/healthcare costs and health insurance in addition to other related factors.

**Results:**

Although the rapid expansion of social health insurance coverage has significantly improved the healthcare utilization among middle-aged and elderly adults, the difference between three schemes is large. Urban Employee Medical Insurance (UEMI) has had a greater effect in improving healthcare utilization than New Cooperative Medical Insurance (NCMI) or Urban Resident Medical Insurance (URMI). Unification of health insurance programs and optimization of health resource allocations should be a practical way to alleviate healthcare utilization inequality across schemes. People having social health insurance spend more on total and out-of-pocket (OOP) healthcare costs than people not covered by social health insurance, suggesting that enrollment in social health insurance induces significant increases in both total and OOP healthcare expenses. UEMI for the urban employed has relatively higher funding criteria and reimbursement rate, which makes the greatest extent to induce increase in healthcare costs. Some demographic or socioeconomic factors significantly affect healthcare utilization and costs among middle-aged and elderly adults.

**Conclusion:**

Our study demonstrates the differences in healthcare utilization and costs between those with and without social health insurance and between those with different health insurance schemes. Policy efforts should further focus on adjusting social health insurance and optimizing healthcare resource allocation in order to enhance effective utilization of healthcare services and control cost increases among middle-aged and elderly adults.

## Background

Improving access to healthcare services for all through universal health coverage is one of main targets of the Sustainable Development Goals (SDGs) [1]. The ultimate goal of social health insurance is to provide affordable, cost-effective, and equitable healthcare for all people [2]. China has conducted a series of health reforms during the past two decades, including expansion of social health insurance coverage and improvement in the healthcare system. Social health insurance reform employed the strategy of universal coverage and has progressed with expanded benefits during the last decade [3]. There are mainly three types of social health insurance schemes in China: 1) Urban Employee Medical Insurance (UEMI); 2) Urban Resident Medical Insurance (URMI); and 3) New Cooperative Medical Insurance (NCMI). The UEMI for those who were employed in the urban sector was formally established in 1998. After that, the NCMI for rural residents and the URMI covering urban residents who had no formal employment were introduced in 2003 and 2007, respectively. These three schemes covered > 95% of the population (about 1.336 billion people) by the end of 2015 [4]. However, healthcare utilization and costs are still very unequal across different sub-populations, and rapid expansion of social health insurance has not reached the universal level of generosity seen in other countries [5].

Many studies indicate that healthcare utilization and costs are influenced by demographic or socioeconomic factors such as age, gender, marital status, education, health insurance, standard of living, and urban residence. Hansen et al. (2012) and Saeed et al. (2013) found that health status, income, health insurance, and other demographic factors are strong determinants of healthcare utilization [6, 7]. Patient factors may be more important than supply factors in explaining the differential use of health services [8]. The most recent studies have focused on the effects of health insurance on healthcare utilization or costs and have shown mixed results. Fang et al. (2012), Kondo et al. (2013) indicated a significant positive correlation between health insurance and healthcare utilization and costs [9, 10]. Gudindon (2014), Grigorakis et al. (2016) showed that health insurance increased the use and costs of inpatient but not outpatient services [11, 12]. Gotsadze et al. (2015) reported that the Medical Insurance Schemes may have improved healthcare utilization and reduced costs [13].

China has made significant improvements in life expectancy over the last two decades due to developments in economy and medical treatment. The economic reform and opening up since 1978 in China created a shift in health systems from “equal access for equal need” to one that is profit-oriented and gradually increased reliance on health insurance and user payment [14]. There are useful studies in which China’s social health insurance over the last several years has been analyzed. Most studies focus only on NCMS, URMI, or UEMI separately. The studies on NCMS are mainly concentrated on the effects of access to NCMS on healthcare utilization and costs. Wagstaff et al. [15] found that enrollment in NCMS increased healthcare utilization but has no effects on out-of-pocket (OOP) costs. Babiarz et al. [16] reported that NCMS has been associated with a significant increase in healthcare utilization in village clinics, and OOP costs have decreased with access to NCMS. Cheng et al. [17] focused on the elderly and found that these participants are more likely to get adequate medical services, but the NCMS has not reduced their OOP spending. Focusing on urban people without access to the UEMI scheme, some studies have examined the effects of URMI on healthcare utilization and costs. Liu and Zhao [18] showed that the URMI scheme has significantly increased the utilization of formal medical services and total healthcare costs but has not reduced OOP costs. Similar results were found by Chen et al. [19]. Zeng et al. [20] showed that enrollment in URMI significantly increased the elderly’s total medical expenses. There are a few studies about the effects of UEMI on healthcare utilization and costs. Huang et al. [21] examined UEMI’s effects on the demand for healthcare services and found that the UEMI has been associated with decreased outpatient medical care utilization and expenditures but not with decreased inpatient care utilization and expenditures. Chen et al. [22] reported that people enrolling in UEMI witnessed a significant increase in the medical expenses compared to those no longer covered by UEMI.

In recent years, the age of the Chinese population has increased. The elderly are more sensitive to healthcare utilization and costs because of the high prevalence of chronic disease, low incomes, and the absence of social security [23]. Despite many studies that have examined health insurance worldwide, most have focused on whole populations rather than specific, vulnerable groups. Furthermore, in China, there still are significant differences in access to services and problems with total and OOP healthcare costs among people having different social health insurance schemes [24]. However, few studies have examined the disparity of the effects of social health insurance on healthcare utilization and costs. In this paper, using national representative survey data in China, we calculated the total amount of healthcare utilization, the prevalence of healthcare underutilization, and healthcare costs among middle-aged and elderly adults; we then estimated the differences in healthcare utilization and costs between those with and without social health insurance and between those with different social health insurance schemes.

## Methods

### Data source

Data were obtained from a nationally representative middle-aged and elderly household survey, the China Health and Retirement Longitudinal Study (CHARLS), which is available online at: http://charls.ccer.edu.cn/en/page/data/2015-charlswave1. This survey, which included 150 counties in 28 provinces, aims to construct a high-quality nationally representative sample of Chinese community-dwelling adults aged > 45 in order to conduct scientific research concerning the elderly [25]. The respondents in this survey include middle-aged and elderly adults ≥45 years in any household.

The sample was drawn in four stages. First, county-level units (counties or urban districts) were sampled directly. These counties cover 28 of 30 provinces in mainland China, other than Tibet. Second, village and community units within county units were chosen with the help of the National Bureau of Statistics, using recently updated village-level population data. The sample used administrative villages in rural areas and neighborhoods in urban areas as primary sampling units (PSUs). Three PSUs were selected within each county-level unit, using probability proportional to size (PPS) sampling, to select a total of 450 PSUs. Third, household units were selected in each PSU. The sampling frame was constructed using Google Earth base maps, and a computer-assisted personal interview program was then used to sample households and to conduct the interviews using portable computers. Specifically, GPS information was collected by photographing at the door of the household. Then, using personal interview program in portable computer, the interviewer interviewed the respondents and filled out each module of the questionnaire. The data was uploaded to the project group through internet at the end of the day. Finally, all age-eligible sample households who were willing to participate in the survey were interviewed.

The main questionnaire includes information on basic demographics, family, health status and functioning, health care and insurance, work, retirement and pension, income, expenditures and assets. With regard to healthcare utilization, monthly outpatient consultations (including face-to-face consultations and home visits) and yearly hospitalizations and dental visits were recorded. Healthcare underutilization was also recorded when asked whether or not to perceive a need for outpatient or inpatient care but did not seek treatment, early dismissal from the hospital and whether or not to see a dentist during the past year. Furthermore, reasons for not seeking outpatient or inpatient care (1. Illness is not serious. 2. Poor. 3. No time. 4. Not willing to go to hospital. 5. Poor quality of hospital. 6. Other) and reasons for early dismissal from the hospital (1. Can’t recover from illness. 2. Poor. 3. Poor quality of hospital. 4. Other) were asked in the questionnaire. Data on total and OOP costs of outpatient, inpatient, medication and dentist were collected by asking how much the total cost and paying out of pocket after reimbursement from healthcare insurance were during the outpatient visit, hospitalization, self-medication or dentist visit.

The total number of enrolled participants was 18,433. Detailed descriptions of the samples are included in Table 1.

**Table 1:** Description of sample

Variable/categories	Prevalence (%)	95% CI		n
Health insurance				
No insurance	4.71	0.0440	0.0502	861
UEMI (Urban employee medical insurance)	12.53	0.1205	0.1301	2289
URMI (Urban resident medical insurance)	7.27	0.0675	0.0780	1329
NCMI (New cooperative medical insurance)	73.99	0.7335	0.7462	13,521
other commercial insurance	1.5	0.0133	0.0168	275
Sex				
Male	47.74	0.4702	0.4846	8800
Female	52.26	0.5154	0.5298	9633
Age				
≤ 50	15.65	0.1513	0.1618	2885
> 50 and ≤ 65	43.13	0.4242	0.4385	7951
> 65	41.21	0.4050	0.4192	7597
Educational level				
Less than lower secondary education	87.09	0.8661	0.8758	16,038
Upper secondary and/or vocational training	10.77	0.1033	0.1122	1984
Tertiary education	2.13	0.0193	0.0234	393
Income level				
Lowest quintile	20.06	0.1930	0.2082	2153
Second quintile	19.94	0.1918	0.2069	2140
Third quintile	20.05	0.1929	0.2081	2152
Fourth quintile	19.95	0.1919	0.2070	2141
Highest quintile	20.00	0.1925	0.2076	2147
Marital status				
Married/cohabiting	81.14	0.1830	0.1943	14,950
Single/divorced/widowed	18.86	0.8057	0.8170	3476
Urban or rural residence				
Urban	37.74	0.3697	0.3851	5699
Rural	62.26	0.6149	0.6303	9401
Health self-reporting				
Excellent	1.37	0.0120	0.0155	241
Very good	10.06	0.0961	0.1050	1764
Good	14.26	0.1374	0.1478	2501
Fair	52.51	0.5177	0.5325	9208
Poor	21.80	0.2119	0.2241	3823

Among the individuals included in the survey, 4.71% had no health insurance, 1.5% had other commercial insurance, nearly 74, 13, and 7% enrolled in NCMI, UEMI, and URMI, respectively. Regarding socioeconomic classification, 41% were > 65 years, approximately 87% had less than lower secondary education, and 40% were in the second or lowest income quintile.

## Data analysis

### Measuring healthcare utilization

The number of outpatient consultations (including face-to-face consultations and home visits), hospitalizations and dental visits during the past year were calculated. We also calculated the prevalence of healthcare underutilization by type of health insurance. Specifically, outpatient care (perceived a need for outpatient care but did not seek outpatient treatment in the past month), inpatient care (perceived a need for inpatient care but were not hospitalized during the past year), early dismissal from the hospital, and dental care (whether or not to see a dentist during the past year) were included. Furthermore, according to the questionnaires, the prevalence of reasons for healthcare underutilization (including reasons for not seeking outpatient or inpatient care, reasons for early dismissal from the hospital) were assessed.

### Healthcare costs

The total and OOP costs covered by different types of health insurance were calculated for each patient. The costs were further divided into medication, outpatient, inpatient, and dental costs. Medication costs included those medications that were purchased at the pharmacy during the past month. Outpatient costs were the value of expenditures of the doctor’s visit for the respondent in the past month. Inpatient costs were the value of hospitalization expenditures for the respondent in the past year. Dental costs included the value of dental care expenditures, including dentures, for the respondent in the past year.

### Statistical analysis

Initially, descriptive statistics were used to show the prevalence of each variable with 95% confidence intervals (CI). Healthcare utilization, including the number of outpatient consultations, the number of hospitalizations, the number of dental visits, mean length of hospital stay, and the prevalence of healthcare underutilization, was then presented by different types of health insurance. Medication, outpatient, inpatient, and dental costs were calculated for different types of samples. Finally, logistic and multiple linear regression models were conducted to evaluate the association between healthcare utilization or healthcare costs and health insurance and other related factors [26]. The regression model can be written as follows:

1 $$ {Y}_i={\sum}_k{\beta}_k{X}_{ki}+{\varepsilon}_i $$

Where *Y*_*i*_ denotes healthcare underutilization (when the subject answered positively to one or more of the following: perceived a need for outpatient care but did not seek outpatient treatment, perceived a need for inpatient care but did not go to the hospital, did not see a dentist for dental care, or early dismissal from the hospital), the total amount of healthcare utilization per year (the number of outpatient consultations + the number of hospitalizations + the number of dental visits) or healthcare costs (total costs and OOP) in a middle-aged and elderly population. *X*_*k*_ denotes healthcare insurance and other related factors: 1) health insurance plan; 2) age; 3) gender; 4) marital status; 5) educational level; 6) income level; 7) self-reported health status, and 8) live in urban or rural. Detailed descriptions of the variables and indicators are included in Table 2.

**Table 2:** Description of dependent and predicting variables

	Categories	Indicators/survey questions
Dependent variable		
Healthcare underutilization		
Outpatient	Reported non-use of outpatient/no reported non-use of outpatient	Questions: Have you been ill in the last month? What the main reason for not seeking medical treatment?
Inpatient	Reported non-use of inpatient/no reported non-use of inpatient	Question: In the past year, did a doctor suggest that you needed inpatient care but you did not get hospitalized?
Dentist	No seen the dentist/ seen the dentist	Question: Have you seen a dentist for dental care in the last year?
Early dismissal from the hospital	Reported dismissal from the hospital/ No reported dismissal from the hospital	Question: Why did you want to leave the hospital before you were recovered?
Reason for health underutilization		
Reason for not seeking outpatient	Illness is not serious/No time/ Hospital quality poor/ other	Question: What’s the main reason for not seeking medical treatment?
Reason for not seeking inpatient	Poor/Not willing to go to hospital/ Hospital quality poor/other	Question: What’s the main reason for not seeking hospitalization?
Reason for early dismissal from the hospital	Can’t recover from illness/ Poor/ Hospital quality poor/ other	Question: Why did you want to leave the hospital before you were recovered?
Healthcare utilization		
The number of outpatient visits		Questions: Which types of medical facilities have you visited in the last month for outpatient treatment? How many times did you visit these types of medical facilities during the last month?
The number of hospitalizations		Question: How many times have you received inpatient care during the last year?
The number of dentist visits		Question: How many times have you received dental care during the last year?
Length of hospital stay		Question: How many nights were you hospitalized?
Healthcare costs		
Total medication costs		Question: What is the approximate total cost of treat yourself during the last month? (Consumed medicine, health supplement, health care equipment etc.)
OOP medication costs		Question: How much did you pay out-of-pocket?
Total outpatient costs		Question: How much did all the outpatient visit cost during the last month?
OOP outpatient costs		Question: How much did you pay out of pocket, after reimbursement from insurance?
Total inpatient costs		Question: What was the total medical cost of hospitalization?(Only include the fees paid to the hospital)
OOP inpatient costs		Question: How much did you pay out of pocket for the hospitalization, after reimbursement from insurance?
Total dentist costs		Question: What was the total medical cost for all the dental care in the last year?
OOP dentist costs		Question: How much did you pay out of pocket, after reimbursement from insurance?
Independent variables		
Health insurance	0 No insurance/1 UEMI/2 URMI/3 NCMI/4 Other	Question: Are you the policy holder/primary beneficiary of any of the types of health insurance?
Sex	Male/Female	
Age in years	≤55/56–65/> 65	
Educational level	Less than lower secondary/upper secondary & vocational training/tertiary	Question: What’s the highest level of education in last wave?
Marital status	Not-married/Married	Question: What is your marital status?
Health self-reported	Excellent/Very good/Good/Fair/Poor	Question: Would you say your health is excellent, very good, good, fair, or poor?
Income level	≤1920¥/1920–10,100¥/10,100–276,223¥/≥27,622–53,520¥/> 53,520¥	Yearly household income divided by number of household numbers; 1st household-member with a weight of 1, all following household members with a weight of 0.5
Live in Urban or Rural	Urban/rural	

## Results

Health care utilization and costs by type of health insurance are included in Tables 3 and 4. Table 3 shows that 29% of respondents who perceived a need for outpatient care did not seek outpatient treatment in the past month. This proportion was highest among the middle-aged and elderly population not covered by social health insurance (43%), second highest among people with URMI (41%), and lowest among people covered by NCMI (28%). The main reason for not seeking outpatient care was “illness is not serious.” The proportion of individuals perceiving a need for inpatient care but not undergoing hospitalization during the past year was approximately 21%, and it was also highest among people not covered by social health insurance (33%). People with URMI demonstrated the second highest prevalence (31%) and the lowest prevalence consisted of people covered by UEMI (14%). The primary reason for not going to the hospital was “poor”. Eighty-three percent of the total respondents did not see a dentist during the past year, which was highest among the population covered by NCMI (84%). Of the total inpatients, 31% left the hospital before recovery, which can be explained mostly by economic reasons (50%). The people not covered by social health insurance demonstrated the highest prevalence (45%), and people covered by UEMI demonstrated the lowest prevalence (27%). The mean number of outpatient visits per month (in patients with at least one outpatient visit) amounted to 2.02 in middle-aged and elderly adults. The corresponding figures were higher among people covered by UEMI than in the other three groups. The highest mean number of hospitalizations per year (in patients with at least one hospitalization) was found among middle-aged and elderly adults covered by URMI (1.78), and the lowest number was 1.37 in people not covered by social health insurance. The mean number of dental visits per year (in patients with at least one dental visit) was 2.6, and people covered by NCMI demonstrated the highest number of dental visits (2.68).

**Table 3:** Healthcare utilization by type of health insurance in middle-aged and elderly adults

	Not covered by social health insurance		UEMI		URMI		NCMI		total		Sign
	Mean	SD	Mean	SD	Mean	SD	Mean	SD	Mean	SD	
Healthcare underutilization											
Outpatient	0.4256	0.4855	0.2915	0.4548	0.4070	0.4921	0.2779	0.4480	0.2909	0.4542	**
Inpatient	0.3321	0.4616	0.1445	0.3520	0.3091	0.4635	0.2116	0.4085	0.2099	0.4073	***
Dentist	0.8427	0.3643	0.7660	0.4235	0.8010	0.3994	0.8436	0.3633	0.8311	0.3746	***
Early dismissal from the hospital	0.4490	0.5025	0.2677	0.4434	0.3404	0.4764	0.3152	0.4648	0.3118	0.4633	**
Reason for not seeking outpatient care											
Illness is not serious	0.5750	0.4975	0.7696	0.4222	0.7339	0.4437	0.6600	0.4739	0.6746	0.4687	
Poor	0.2125	0.4117	0.0366	0.1884	0.1048	0.3076	0.1505	0.3577	0.1365	0.3435	
No time	0.0375	0.1912	0.0105	0.1021	0.0242	0.1543	0.0265	0.1606	0.0249	0.1560	
Hospital quality poor	0.0375	0.1912	0.0419	0.2009	0.0484	0.2155	0.0579	0.2336	0.0542	0.2266	
Other	0.1375	0.3465	0.1414	0.3493	0.0887	0.2855	0.1050	0.3067	0.1097	0.3126	
Reason for not seeking inpatient care											
Poor	0.6667	0.4787	0.3273	0.4714	0.5606	0.5001	0.6120	0.4876	0.5764	0.4944	
Not willing to go to hospital	0.2121	0.4151	0.3364	0.4746	0.2273	0.4223	0.2283	0.4200	0.2405	0.4276	
Hospital quality poor	0.0000	0.0000	0.0818	0.2753	0.0606	0.2404	0.0336	0.1804	0.0401	0.1963	
Other	0.1212	0.3314	0.2545	0.4376	0.1515	0.3613	0.1261	0.3321	0.1430	0.3503	
Reason for early dismissal from the hospital											
Can’t recover from illness	0.1053	0.3153	0.1899	0.3947	0.0714	0.2623	0.1561	0.3634	0.1549	0.3622	
Poor	0.5789	0.5073	0.3038	0.4628	0.5000	0.5092	0.5294	0.4997	0.4982	0.5004	
Hospital quality poor	0.0000	0.0000	0.0759	0.2666	0.0000	0.0000	0.0566	0.2313	0.0546	0.2274	
Other	0.3158	0.4776	0.4304	0.4983	0.4286	0.5040	0.2579	0.4380	0.2923	0.4552	
Healthcare utilization											
The number of outpatient visits	1.75	2.01	2.32	3.39	1.93	2.04	2.11	2.64	2.02	2.42	***
The number of hospitalizations	1.37	0.82	1.59	1.58	1.78	2.22	1.51	1.13	1.53	1.27	*
The number of dental visits	2.57	2.20	2.36	2.53	2.36	1.91	2.68	3.94	2.60	3.55	**
Length of hospital stay	12.49	13.58	13.92	13.86	13.32	23.35	12.05	13.50	12.42	14.19	***

Not covered by social health insurance includes the middle-aged and elderly population who did not enroll in any social health insurance (including UEMI, URMI, or NCMI) but possibly has commercial health insurance. Calculations were weighted using individual sampling weights and adjusted for household and individual responses

Note: *** implies *p*-value < 0.001, ** indicates *p*-value < 0.01, * implies *p*-value < 0.05

**Table 4:** Healthcare costs (in dollar terms) by type of health insurance in middle-aged and elderly adults

	Not covered by social health insurance		UEMI		URMI		NCMI		total		Sign
	Mean	SD	Mean	SD	Mean	SD	Mean	SD	Mean	SD	
Medication costs (per month)											
Total	27.69	45.99	46.18	88.89	34.78	70.58	28.43	75.44	31.32	76.37	***
OOP	27.53	45.43	44.41	86.04	34.09	69.60	27.15	73.14	29.86	74.07	***
Outpatient costs (per month)											
Total	19.59	112.73	63.75	649.14	61.86	685.73	43.25	1087.34	45.78	993.53	**
OOP	17.66	98.67	37.53	619.90	36.78	381.37	26.21	247.90	27.60	323.72	*
Inpatient costs (per year)											
Total	165.51	1436.21	391.73	1787.08	235.41	1292.73	185.51	1013.36	214.04	1182.68	***
OOP	113.88	951.67	157.39	838.62	138.25	917.84	117.28	705.91	123.89	750.83	**
Dentist costs (per year)											
Total	12.35	51.68	26.62	106.15	15.32	57.84	11.39	66.85	13.62	72.27	***
OOP	12.34	51.67	24.24	101.02	15.24	57.69	11.04	58.44	13.02	65.29	***
Total healthcare costs (per year)											
Total	559.63	2052.96	1482.58	8104.24	1227.89	8570.99	875.85	13,093.33	958.43	12,010.06	*
OOP	482.58	1611.43	910.94	7421.37	831.08	4856.40	596.74	3262.60	644.77	4091.15	***

Not covered by social health insurance includes the middle-aged and elderly population who did not enroll in any social health insurance plan (including UEMI, URMI, or NCMI), but possibly have commercial health insurance. Calculations were weighted using individual sampling weights and adjusted for household and individual responses

Note: *** implies *p*-value < 0.001, ** indicates *p*-value < 0.01, * implies *p*-value < 0.05

Table 4 shows the medication, outpatient, inpatient, and dental costs and total healthcare costs in middle-aged and elderly adults by type of health insurance. Mean total healthcare costs were significantly higher in the people covered by social health insurance compared to those who were not covered. Among the people covered by social health insurance, those who enrolled in the UEMI demonstrated the highest total healthcare costs and reimbursement rates. According to Table 4, mean reimbursement proportions of outpatient and inpatient costs were 40.1 and 43.1%, respectively, and these were higher among people covered by UEMI (41.2%, 59.8%) than people covered by URMI (40.5%, 41.3%), or NCMI (39.4%, 42.2%). Medication and dental costs were almost entirely paid by OOP-payments in middle-aged and elderly adults with all types of health insurance.

The results from the logistic regression model on healthcare underutilization are presented in Table 5. Social health insurance was significantly associated with healthcare underutilization. Middle-aged and elderly adults with social health insurance were less likely to report underutilization in comparison with individuals without social health insurance (OR = 0.593, 0.892, 0.757). Furthermore, individuals covered by UEMI demonstrated the lowest likelihood of underutilization (OR = 0.593), and people with NCMI demonstrated the second lowest likelihood of underutilization (OR = 0.757). The multivariate model shows a lower likelihood of underutilization of healthcare among middle-aged and elderly adults (age > 65), those who are married or cohabiting, and those living in urban areas. With respect to health status, individuals who self-reported “fair” were more likely to report underutilization of all types of social health insurance. In contrast with the lowest income quintile, the second to highest income group demonstrated the lower likelihood of healthcare underutilization, and the likelihood decreased with increasing income quintile.

**Table 5:** Logistic regression model on healthcare underutilization in middle-aged and elderly adults

Variables/categories	Odds Ratio	Std. Err.	Odds Ratio	Std. Err.	Odds Ratio	Std. Err.
Health insurance; ref.: Not covered by social health insurance						
UEMI	0.593^***^	0.102				
URMI			0.892^**^	0.111		
NCMI					0.757^**^	0.113
Female, ref.: Male	0.829	0.121	1.021	0.186	1.099	0.069
Age in years; ref.:<=50						
> 50 and <=65	0.642^**^	0.121	0.664^*^	0.142	0.800^**^	0.061
> 65	0.489^**^	0.102	0.580^**^	0.150	0.696^***^	0.065
Educational level; ref.: Less than lower secondary education						
Single/divorced/widowedUpper secondary & vocational training	1.013	0.183	1.532	0.376	0.892	0.127
Single/divorced/widowedTertiary education	0.878	0.303	0.800	0.928	1.087	0.818
Marital status; ref.:						
Single/divorced/widowed						
Married/cohabiting	0.722^*^	0.140	0.845	0.194	0.715^***^	0.058
Health status (self-reported); ref.: Excellent						
Very good	1.119^***^	0.059	1.207^***^	0.095	1.129^***^	0.027
Good	1.396^**^	0.128	1.276^**^	0.120	1.212^***^	0.035
Fair	1.597^*^	0.089	1.411^**^	0.138	1.381^***^	0.049
Poor	1.131	0.315	0.636	0.222	0.715	0.097
Income level; ref.: Lowest quintile						
Second quintile	0.734^***^	0.091	0.838^**^	0.104	1.121	0.098
Third quintile	0.722 ^**^	0.121	0.841^**^	0.114	1.038	0.095
Fourth quintile	0.651^*^	0.129	0.690^***^	0.045	0.921^*^	0.133
Highest quintile	0.532^*^	0.130	0.380^**^	0.124	0.899^**^	0.097
Live in Urban or Rural; ref.: urban	1.607^**^	0.107	1.600^*^	0.143	1.802^***^	0.055

Note: *** implies *p*-value < 0.001, ** indicates *p*-value < 0.01, * implies *p*-value < 0.05

Estimates were weighted using individual sampling weights and adjusted for household and individual responses

Table 6 shows the results of the multiple regression model on the total amount of healthcare utilization in middle-aged and elderly adults. These results indicate a significant positive correlation between social health insurance and the total amount of healthcare utilization, which means that having social health insurance increases the amount of healthcare utilization. This effect was estimated to be the greatest among individuals with UEMI and the second greatest among individuals with NCMI. Elderly people were significantly associated with a higher healthcare utilization amount. This was true for the age groups 50–65 years and > 65 years when compared with individuals ≤ 50 years. The total amount of healthcare utilization was significantly higher in women than men among individuals with URMI and NCMI. In contrast with the lowest income quintile, the total amount of healthcare utilization was significantly increased in the second highest and highest income groups for all types of health insurance, and this effect was greatest among people covered by UEMI under the same income quintile. With regard to health status, the results exhibited a significant increase of healthcare utilization in groups that self-reported “poor,” “fair,” or “good” in contrast with the group self-reporting “excellent.”

**Table 6:** Multiple regression model on total amount of healthcare utilization in middle-aged and elderly adults

Variables/categories	Coef.	Std. Err.	Coef.	Std. Err.	Coef.	Std. Err.
Health insurance; ref.: Not covered by social health insurance						
UEMI	0.258^***^	0.066				
URMI			0.129^*^	0.066		
NCMI					0.188^***^	0.039
Female, ref.: male	0.028	0.052	0.312^*^	0.135	0.096^*^	0.041
Age in years;ref.:<=50						
> 50 and <=65	0.183^**^	0.059	0.044	0.067	0.068^**^	0.024
> 65	0.277^***^	0.073	0.324^*^	0.161	0.139^***^	0.031
Educational level; ref.: Less than lower secondary education						
Upper secondary and vocational training	−0.025	0.063	−0.133^*^	0.052	−0.023	0.038
Tertiary education	−0.015	0.119	0.343	0.319	0.269	0.281
Marital status; ref.:						
Single/divorced/widowed						
Married/cohabiting	0.056	0.068	0.020	0.078	−0.041	0.030
Health status (self-reported); ref.: Excellent						
Very good	0.274^**^	0.102	0.251^*^	0.125	0.191^***^	0.024
Good	0.234^*^	0.115	0.289^*^	0.128	0.212^***^	0.032
Fair	0.093	0.103	0.257^*^	0.114	0.267^***^	0.049
Poor	0.687^***^	0.123	0.749^***^	0.134	0.697^***^	0.054
Income level; ref.: Lowest quintile						
Second quintile	0.099^***^	0.023	0.078^**^	0.029	0.016^**^	0.006
Third quintile	0.107^**^	0.041	0.055^**^	0.018	0.015^***^	0.003
Fourth quintile	0.143^**^	0.053	0.050^*^	0.022	0.032^**^	0.012
Highest quintile	0.161^***^	0.027	0.079^***^	0.017	0.010^**^	0.004
Live in Urban or Rural; ref.: urban	−0.094	0.060	−0.064	0.065	−0.071^**^	0.024

Estimates were weighted using individual sampling weights and adjusted for household and individual responses

Note: *** implies *p*-value < 0.001, ** indicates *p*-value < 0.01, * implies *p*-value < 0.05

Table 7 reports the results of the multiple regression model on total healthcare costs in middle-aged and elderly adults. The social health insurances were significantly associated with total healthcare costs. Those people covered by social health insurance spend more on healthcare than people not covered by social health insurance, and this impact was estimated to be greater among people covered by UEMI in comparison with people with URMI or NCMI. Furthermore, results revealed a significant correlation between some demographic or socioeconomic factors and total healthcare costs. Specifically, total healthcare costs were significantly higher in men than women who were covered by all types of social health insurance schemes. Elderly people > 50 years spent more on healthcare than people ≤ 50 years, and this effect was estimated to be greatest among individuals covered by UEMI. The individuals who self-reported health statuses of “good” “fair,” or “poor” were associated with higher total costs, whereby costs increased with declining health conditions. Under the same health conditions, this effect was greatest among people with NCMI. Compared with the lowest income quintile, the second to highest income quintile was significantly associated with higher total healthcare costs. This effect was estimated to be greater among individuals with UEMI or URMI rather than NCMI.

**Table 7:** Multiple regression model for total healthcare costs in middle-aged and elderly adults

Variables/categories	Coef.	Std. Err.	Coef.	Std. Err.	Coef.	Std. Err.
Health insurance; ref.: Not covered by social health insurance						
UEMI	1.507^***^	0.266				
URMI			1.033^***^	0.275		
NCMI					0.920^***^	0.174
Female, ref. male	− 0.406^*^	0.186	− 0.556^*^	0.239	− 0.296^***^	0.077
Age in years; ref.:<=50						
> 50 and <=65	0.931^***^	0.260	0.576^*^	0.270	0.374^***^	0.097
> 65	1.202^***^	0.288	0.922^**^	0.358	0.343^**^	0.118
Educational level; ref.: Less than lower secondary education						
Upper secondary & vocational training	0.331	0.223	0.442	0.330	0.181	0.153
Tertiary education	0.850^*^	0.370	1.815	1.358	1.505	0.924
Marital status; ref.:						
Single/divorced/widowed						
Married/cohabiting	−0.073	0.251	0.145	0.316	0.037	0.109
Health status (self-reported); ref.: Excellent						
Very good	0.649	0.512	1.265^*^	0.602	1.793^***^	0.240
Good	0.977^*^	0.484	0.928	0.581	2.150^***^	0.226
Fair	2.490^***^	0.449	2.377^***^	0.507	3.279^***^	0.210
Poor	4.543^***^	0.477	4.323^***^	0.541	5.118^***^	0.220
Income level; ref.: Lowest quintile						
Second quintile	0.773	0.564	0.829	0.503	0.319^**^	0.112
Third quintile	0.842^*^	0.406	0.855^*^	0.418	0.551^***^	0.113
Fourth quintile	1.253^**^	0.432	0.999^*^	0.448	0.469^***^	0.121
Highest quintile	1.047^*^	0.435	1.031^*^	0.449	0.428^***^	0.129
Live in Urban or Rural; ref.: urban	−0.073	0.236	0.058	0.280	−0.276^***^	0.086

Estimates were weighted using individual sampling weights and adjusted for household and individual responses

Note: *** implies *p*-value < 0.001, ** indicates *p*-value < 0.01, * implies *p*-value < 0.05

In Table 8, we estimate health insurance and other correlates of OOP healthcare costs in middle-aged and elderly adults. These results indicate a significant positive correlation between social health insurance schemes and OOP healthcare costs, which means that for people with social health insurance, OOP healthcare costs will be greater than for those without these schemes. This can be interpreted as a result of the higher likelihood of healthcare underutilization among middle-aged and elderly adults without social health insurance (Table 5) and lower reimbursement rates of Chinese social health insurance (Table 4). The results also revealed a significant correlation between demographic or socioeconomic factors and OOP healthcare costs, and these estimation effects were similar to those seen in Table 7.

**Table 8:** Multiple regression model for OOP healthcare costs in middle-aged and elderly adults

Variables/categories	Coef.	Std. Err.	Coef.	Std. Err.	Coef.	Std. Err.
Health insurance; ref.: Not covered by social health insurance						
UEMI	1.128^***^	0.267				
URMI			0.937^***^	0.274		
NCMI					0.882^***^	0.173
Female, ref. male	−0.556^**^	0.188	−0.713^**^	0.238	−0.304^***^	0.075
Age in years; ref.:<=50						
> 50 and <=65	0.818^**^	0.263	0.578^*^	0.296	0.341^***^	0.096
> 65	0.935^***^	0.293	0.632	0.364	0.252^*^	0.116
Educational level; ref.: Less than lower secondary education						
Upper secondary & vocational training	0.307	0.229	0.435	0.336	0.189	0.152
Tertiary education	0.634	0.406	1.915	1.290	1.623	0.919
Marital status; ref.:						
Single/divorced/widowed						
Married/cohabiting	−0.011	0.261	0.190	0.315	0.075	0.106
Health status (self-reported); ref.: Excellent						
Very good	0.681	0.514	1.454^*^	0.601	1.711^***^	0.236
Good	1.109^*^	0.484	0.991^*^	0.477	2.100^***^	0.222
Fair	2.474^***^	0.448	2.607^***^	0.509	3.201^***^	0.207
Poor	4.143^***^	0.480	4.521^***^	0.542	4.951^***^	0.217
Income level; ref.: Lowest quintile						
Second quintile	0.681	0.565	0.682	0.501	0.291^**^	0.110
Third quintile	0.716	0.452	0.591	0.458	0.522^***^	0.111
Fourth quintile	1.059^**^	0.406	0.799^*^	0.417	0.440^***^	0.120
Highest quintile	0.646^*^	0.340	0.700^*^	0.389	0.354^**^	0.128
Live in Urban or Rural; ref.: urban	−0.040	0.238	0.098	0.279	−0.271^***^	0.085

Estimates were weighted using individual sampling weights and adjusted for household and individual responses

Note: *** implies *p*-value < 0.001, ** indicates *p*-value < 0.01, * implies *p*-value < 0.05

## Discussion

China has conducted a series of health reforms during the past two decades, and the insurance coverage is almost universal [4]. However, few studies have examined the disparity of the effects of social health insurance on healthcare utilization and costs, especially among vulnerable groups. This study, focusing on middle-aged and elderly people, was conducted in order to analyze the differences in healthcare utilization and costs between those with and without social health insurance and between different types of social health insurance.

In this study, we found that 29% of respondents who perceived a need for outpatient care did not seek outpatient treatment and 21% of respondents who perceived a need for inpatient care did not seek inpatient treatment, and these proportions are highest among the middle-aged and elderly population not covered by health insurance (43%, 33%). Furthermore, the people not covered by social health insurance demonstrate the lowest mean number of outpatient visits and hospitalizations (Table 3). The regression results shown in Tables 5 and 6 further confirm that people with social health insurance are less likely to report underutilization and are significantly associated with a higher amount of healthcare utilization. These findings indicate that enrollment in social health insurance improves healthcare utilization among middle-aged and elderly adults, similar to that seen in existing studies [6–8, 10, 12]. Among the people covered by social health insurance, those with URMI demonstrate the highest prevalence of outpatient and inpatient underutilization (Table 3), and middle-aged and elderly adults with UEMI demonstrate the lowest likelihood of underutilization and the greatest effect of health insurance on the amount of healthcare utilization (Tables 5 and 6). This indicates that although the rapid expansion of health insurance coverage has improved the availability of health services, the differences in healthcare utilization between the three schemes is large. UEMI has had a greater effect on improving healthcare utilization than NCMI or URMI, which may be caused by different patterns in health insurance policy design and irrational allocation of health resources between urban and rural areas in China [27]. Unification of health insurance schemes and optimization of health resource allocation could be a practical way to alleviate this inequality in healthcare utilization.

Total healthcare costs were significantly higher in the people covered by social health insurance compared to those who were not covered (Table 4). The regression results also indicate that people with social health insurance spend more on total healthcare costs than people not covered by social health insurance (Table 7). This finding is similar to other published studies [9, 13, 16, 18–20, 22]. However, our analysis shows that people with social health insurance also spend more on OOP healthcare costs than people not covered by social health insurance (Tables 4 and 8); this finding is similar to that found by Grigorakis et al. (2016), which indicated that enrolling in health insurance actually induced a significant increase in OOP health costs among middle-aged and elderly adults. This may be caused by a higher likelihood of healthcare underutilization among middle-aged and elderly adults without social health insurance, which reduces the demand and expenditure on health services, and the lower reimbursement rate of social health insurance in China, which produces relatively high OOP healthcare costs [28]. Furthermore, we found that middle-aged and elderly adults with UEMI had the highest healthcare costs and showed the greatest effects of health insurance on costs increase (Tables 4, 7, and 8). This finding reflects the differences in policy design in social health insurance, differences in population characteristics between middle-aged and elderly adult groups, and inequity in health resource allocation in China [29]. UEMI for the urban employed, who have a stable source of income and better accessibility to health services, has relatively higher funding criteria and reimbursement rates in comparison with URMI or NCMI [30]. Therefore, the greatest benefit seems to be to promote healthcare utilization and induce healthcare cost increases (both total healthcare costs and OOP costs).

Other demographic or socioeconomic factors associated with healthcare utilization and costs include age, gender, marital status, health status, living standards, and urban residence. Specifically, older people are significantly associated with lower likelihood of underutilization of healthcare, higher number of healthcare utilization and healthcare costs. This finding is similar to those in most of the previously conducted studies; chronic disease prevalence is higher in the older group, which stimulates more healthcare utilization and costs [6, 7, 9–13]. Those who are married or living in urban areas demonstrate a lower likelihood of healthcare underutilization. Being single is an obstacle to the utilization of health services. Married patients are more likely to seek treatment in regular medical institutions because their spouses may timely urge or take them to a high level medical institution to acquire needed medical care [31]. Compared to urban residents, the lower income of rural residents restricts their effective utilization of health services in China [29]. Therefore, policy effort should further focus on the rural elderly adults or the single to promote effective utilization of health services. We found an inverse association between gender and healthcare utilization and costs. While the total amount of healthcare utilization is significantly higher among women than men, healthcare costs are significantly higher among men than women. This finding is similar to van den Bussche et al. [32]; differences between men and women in the severity of illness and in the health behavior patterns might account for this discrepancy. High-income groups demonstrate a lower likelihood of healthcare underutilization. Meanwhile, healthcare utilization and costs increased with declining health status. These findings indicate that policies aimed at reducing inequality in healthcare utilization must consider economic factors and health status among middle-aged and elderly adults such as implementing strategies that increase income among the low-income elderly, narrow the income gap, and promote effective use of health services according to different health status [33].

Some limitations of our study must be acknowledged. We evaluated healthcare underutilization only among middle-aged and elderly adults perceiving a need for healthcare but did not seek treatment, and did not take into account those not perceiving a need for healthcare. Some patients objectively need healthcare, but they do not perceive or understand that need. Therefore, the prevalence of healthcare underutilization might have been underestimated to some extent. In addition, our study findings are likely to be influenced by additional factors, which are not included in the claims data; these include severity of illness or patients’ social settings. Finally, using cross-sectional data does not allow for any causal conclusions to be drawn.

## Conclusions

Although the rapid expansion of social health insurance coverage in China has significantly improved healthcare utilization and costs among middle-aged and elderly adults, the differences between the three schemes are large. Individuals with UEMI had the highest healthcare costs and demonstrated the greatest effect of health insurance on healthcare utilization and expenses increases. The government should further adjust social health insurance schemes and optimize the allocation of health resources to alleviate the inequality across schemes and enhance effective utilization of healthcare services.

## Abbreviations

CHARLS: China Health and Retirement Longitudinal Study
CI: Confidence intervals
NCMI: New Cooperative Medical Insurance
OOP: Out-of-pocket
PPS: Probability proportional to size
PSUs: Primary sampling units
SDGs: Sustainable Development Goals
UEMI: Urban Employee Medical Insurance
URMI: Urban Resident Medical Insurance
